# The Gates Foundation’s network diplomacy in European donor countries

**DOI:** 10.1186/s12992-025-01112-9

**Published:** 2025-04-24

**Authors:** Antoine de Bengy Puyvallée, Katerini Tagmatarchi Storeng, Simon Rushton

**Affiliations:** 1 Centre for Development and the Environment, University of Oslo, Postboks 1116 Blindern, Oslo, 0317 Norway; 2 University of Sheffield, Sheffield, UK

**Keywords:** Gates Foundation, Network diplomacy, Power, Political advocacy, Influence, Philanthropy, Europe, France, Germany, United Kingdom

## Abstract

The Gates Foundation is the most influential private philanthropic foundation in global health and development. This article examines how the Foundation has developed an unparalleled capacity to rally other donors to its priorities, which include the development and distribution of technological tools to reduce the burden of infectious disease and child mortality in the world’s most impoverished regions. Using publicly available data, the article analyses the Gates Foundation’s strategic engagement in Europe, focusing on its bureaucratic presence, government relations, and grant-making in its three European focus countries: the United Kingdom (UK), Germany and France. It highlights that, since 2010, the Gates Foundation has built a bureaucratic infrastructure akin to a diplomatic service, establishing country offices in London and Berlin alongside representation in Paris, Brussels and Stockholm. Through regular engagement with elected officials and bureaucrats in these nations, the Foundation has forged many strategic partnerships, effectively leveraging European states’ diplomatic power in wider political forums and alliances. Moreover, the Foundation has disbursed billions in grants to recipients in the UK, Germany, and France to advance research and innovation on its priority health issues, implement programs in poor countries, and develop policy and advocacy related to global health and development. Combined, these efforts have contributed to securing substantial and recurrent government co-investment in Gates-supported initiatives. The article proposes that the Foundation exercises a form of ‘network diplomacy’ that entails building and maintaining wide networks across European societies with the aim of aligning donor governments’ overseas development assistance and policies with the Foundation’s strategic objectives in global health and development. The concept of network diplomacy offers a new perspective on how the Gates Foundation has consolidated and expanded its transnational political presence through an approach that is strategic, bureaucratised and institutionalised, rather than simply a product of its financial might. The findings amplify existing concerns regarding the sway of private foundations over public policy, their impact on democratic accountability and governance in donor states, and the resultant implications for the Foundation’s intended beneficiaries in low- and middle-income countries.

**Supplementary Information:**

The online version contains supplementary material available at 10.1186/s12992-025-01112-9.

## Introduction

The Gates Foundation, formerly the Bill & Melinda Gates Foundation, is by far the largest private philanthropic foundation in global health and development. Among its main aims is to reduce inequalities in health by developing new vaccines, medicines and other technological tools to reduce the burden of infectious disease and the leading causes of child mortality in the world’s poorest countries. Having distributed nearly 80 billion dollars in grants over its first 25 years, in 2024, it was the third largest contributor of development assistance for health, surpassed only by the USA and Germany [1]. This has granted the Foundation significant standing in international affairs, sometimes akin to that of a sovereign state. In 2005, Bill Gates was the first non-state actor to address the World Health Assembly, the World Health Organization’s (WHO) governing body. Additionally, Foundation representatives are regular participants at major global summits, alongside governments.

The Gates Foundation exemplifies a new approach to philanthropy, variously described as ‘venture philanthropy’ [2] and ‘philanthrocapitalism’ [3–5]. These terms describe the ambition to transform society by applying business approaches to public policy and harnessing the capitalist system to address social problems [6]. Bill Gates envisions such philanthropy as reliant on leveraging “philanthropic partnerships” with both state and other non-state actors. He has called his approach “catalytic philanthropy,” explaining that “philanthropy’s role is to get things started” by using the Foundation’s funds to shape markets and stimulate action by business and governments in favour of the poor [7].

By implying that change is almost magically triggered by philanthropy, Bill Gates’ notion of catalytic philanthropy downplays the immense agency and influence the Foundation wields to drive change. By contrast, our analysis draws attention to how the Gates Foundation works strategically to organise, sustain and expand its political presence globally [8] and to influence sovereign donors and other social actors to support its approach. While often associated with the private philanthropy of Bill and Melinda Gates and Warren Buffett, the Foundation has developed into a sophisticated transnational organization employing over 2000 staff who collaborate with partners in over 130 countries globally “to address the issues we care about and drive change” [9]. Besides its Seattle headquarters, the Foundation has offices in Washington DC, London, Berlin, Delhi, Beijing, Abuja, Addis Ababa, Johannesburg, Dakar and Nairobi that have been likened to embassies [8]. In addition to its grants to research, innovation and development programmes, it allocates millions — US$ 328 million in 2023 alone globally — in advocacy and policy grants that serve “to build strategic relationships and promote policies that will help advance our work” [10]. Over time, it has developed what Birn [3, p10] has described as “an extraordinary capacity to marshal other donors to its efforts,” as evidenced by their repeated co-investments in initiatives like Gavi, the Vaccine Alliance.

In this article, we show that the concept of ‘network diplomacy’ is helpful for grasping how the Foundation uses a variety of strategies and practices to engage governments and shape wider policy environments in pursuit of its objectives. Network diplomacy has been conceptualised as “a non-hierarchical type of interaction between nations and non-state actors, including negotiations and soft power techniques, with the aim of addressing global problems” [11, p158]. The practice extends nation states’ traditional diplomacy by strategically developing relationships with a diverse array of non-state actors within a country [11–13]. Network diplomacy also involves a public diplomacy strategy designed to maintain, strengthen and expand networks by directly engaging with the public and relevant stakeholders through media and social media platforms [12]. The emergence of this term reflects an acknowledgement that nation states are no longer the sole influential entities in global politics, if indeed they ever were. In a ‘networked’ world order, states and their diplomatic services can significantly influence policy by working with non-state actors [13, 14]. Scholars have exemplified the power of network diplomacy by showing how traditionally less influential states form coalitions with civil society organisations to achieve change, such as an international ban on landmines [15].

Traditionally applied to studies of state diplomacy, we show in this article that powerful private entities like the Gates Foundation also engage in a form of network diplomacy within the global health and development field, which is characterised by a complex network of state and non-state actors that operates both within and across national boundaries. Network diplomacy can facilitate the development of network power, defined as the ability to harness the network’s resources to achieve policy goals [13, 16, 17]. This network power effectively aligns a broad spectrum of stakeholders around a unified vision and common objectives. As the network expands, a preferred policy option gradually becomes dominant, which increasingly appears consensual to stakeholders and the public, leading to the gradual marginalization and elimination of other policy alternatives [18].

This article presents the first analysis of the Gates Foundation’s strategic deployment of network diplomacy to cultivate and maintain partnerships in donor countries. Our focus is on its endeavours in Europe — a region that attracts two-thirds of the Foundation's transnational funding (i.e. money spent outside of the United States), yet remains understudied. The Foundation sees European governments as some of its most important strategic partners because Europe accounts for more than half of the world’s official development assistance (ODA) [19], which the Foundation seeks to leverage.

Empirically, we examine the Foundation’s activities in its three European “focus countries” (France, Germany and the UK) and examine three components of its network diplomacy: first, its territorial and bureaucratic expansion in Europe; second, its direct government relations activities; and third, the pattern of its grant-giving to various recipients between 2000 and 2024, zooming in on its advocacy and policy grants that contribute to shaping both the domestic and international policy environments and public discourse within which the donor governments operate. We then discuss how the Gates Foundation’s practices expand our understanding of the concept of network diplomacy and conclude that our findings underscore the need for critical attention to private foundations’ influence over public policy and their challenge to democratic accountability and governance across world regions.

## Background

The Gates Foundation has reinforced the historical trend of US-based philanthropic foundations (e.g., the Rockefeller and Ford Foundations) as important agents in world politics [20, 21]. However, today’s philanthropic sector differs in sheer size, and in the breath of its influence across society [22]. With the Gates Foundation in the lead, the global philanthropic sector is increasingly dominated by mega foundations that disburse enormous grants from individual or corporate donors seeking to dramatically improve the world. The Gates Foundation, the Danish Novo Nordisk Foundation and the British Wellcome Trust are the biggest in terms of endowment size, with the Mastercard and Ford Foundations among the top ten spenders in the development sector [23].

Among these, the Gates Foundation has had by far the most marked influence on the development of global governance, being actively engaged in efforts to create new global institutions, shape policies, and set agendas [20, 24, 25]. For example, the Foundation played an important role in the establishment of new public-private partnerships at the start of the Millennium Development Goal era, such as the global health initiatives Gavi, the Vaccine Alliance and the Global Fund to Fight AIDS, Tuberculosis and Malaria [20]. These public–private partnerships are organised according to private sector management principles of efficiency and innovation, and joint-decision making between public and private stakeholders [26–28]. Nearly 25 years after they were established, the Gates Foundation continues to hold a seat on the Boards of both organisations. It also wields significant influence over the WHO as the organisation’s second largest donor, providing earmarked funding to specific projects [29] and shaping policy processes through the secondment of staff and consultants [30]. All of this has raised concerns about its lack of legitimacy and accountability (e.g. [31–33]), with many questioning whether it is right that a private foundation should wield so much power on the global stage.

Previous studies have often approached the Gates Foundation as a discrete actor exercising power over others and have analysed the different sources of its power. For example, scholars have argued that the Foundation wields power through its considerable financial clout, by drawing on its recognized in-house expertise and ability to enlist external experts to shape policy agendas [34–36], and through its moral authority that derives partly from its claims of doing good in the world and partly from its status as non-state “independent” actor [8, 37]. Others have identified the institutional power the Foundation derives from its representation and decision-making power in major global health organisations [38, 39], and argued that the charismatic authority of Bill and Melinda Gates opens the doors of high-level decision-makers [32]. The foundation is also recognised as having significant epistemic power, contributing to a privileging of technological solutions and global metrics over structural socio-economic drivers of global health and development and locally relevant health data across global health research and policy [34, 35, 40–42]. By playing a critical role in the production, analysis, interpretation and dissemination of knowledge, the Gates Foundation — akin to other private foundations such as Bloomberg Philanthropies — practices a form of ‘knowledge philanthropy’ which has further entrenched its involvement in global governance [43].

The Gates Foundation has also been criticised for creating parallel structures in low- and middle-income countries [42, 44, 45]. Over time, however, there has been a shift in the Foundation’s attitude to working with states and multilateral organisations. From an initial preference for a “strategy of isolation” [24, p1104], stemming from a distrust for governments [33, p147] and organisation like the WHO [46, p706], the Foundation has embraced engagement with them. According to Partzsch & Fuchs [47, p364], this was the result of a gradual understanding of “how little change a single foundation can make (even if it is the world’s largest foundation).”

Studies of the Gates Foundation’s interactions with states in the context of India [48] and Tanzania [45] highlight the complex relationships between the Foundation and public authorities, which are shaped both through direct contacts and negotiations, but also indirectly through third-party funding and behind-the-scenes influence. However, little research to date has examined in detail how the Foundation works to shape major donor states’ global health and development policies outside of the US.^1^ Fejerskov, one of the few notable exceptions to this, argues that the opening of the Foundation’s London office provided easy access to European governments, enabling “funding collaboration or pressure from the foundation [to] sustain donor support for aid and for the foundation’s priorities” [8, p172]. Moreover, he claims that the local offices in other world regions “provide a point of entrance to diplomatic relations…and combine grantee relations with maintaining a 180 structured dialogue with relevant governments”, [8, p172]. This is an important starting point for our investigation because it suggests a strategy for engaging with donor governments around the world that rests not only on financing or the “celebrity diplomacy” [49] of Bill Gates himself, but an approach that is institutionalised and bureaucratised. 

Our analysis of the Gates Foundation’s activities in Europe is based on publicly available information collated from a wide variety of sources (see ‘Methods’ below for full details). To describe its territorial and bureaucratic expansion in Europe, we draw on the Gates Foundation website and online professional profiles. To describe its government relations, we analyse i) official meeting records and other evidence of meetings between Foundation staff and key government ministers/officials; ii) official partnership agreements/memoranda of understanding (MoUs) with governments; and iii) online evidence of Foundation staff’s participation in major global health and development summits. To analyse its grant-making patterns in Europe, we relied on the Foundation’s publicly available grant database. The analysis builds on our previous research on the ​​Foundation's role as a member of various partnerships (eg. [26, 28, 42]).

## Results

### Territorial and bureaucratic expansion

The Gates Foundation’s strategic relationship with Europe has involved both territorial and bureaucratic expansion over the past fifteen years, including in the United Kingdom, Germany, and France, which the Foundation describes as “highly engaged multilateral partners” [50]. This reflects the fact that they are major donors and partners with the Gates Foundation in initiatives like Gavi and The Global Fund, and also that they convene broader policy networks, diplomatic initiatives and innovation processes with which the Foundation seeks to engage.

The Foundation opened its first European office in the UK in 2010 to support its expanding activities in Europe, as well as the Middle East, and East Asia [51]. According to Joe Cerrell, who set up and still manages the London office, “We established the London office…because of the UK’s long-standing reputation and influence in international development” [52]. The Foundation also points to the UK’s role as an active partner and funder across most of the Foundation’s focus areas, its major role in global health and development, science and diplomacy [52], and that London regularly hosts “major global summits to secure political commitment and funding for urgent issues,” such as vaccine delivery, nutrition, family planning, and malaria control [51].

Following the UK’s vote to withdraw from the European Union, the Foundation opened a Berlin office in 2018, underscoring Germany's rising leadership in global health and the Foundation's longstanding collaboration with both the German Federal Government and civil society [53]. Like the UK, Germany was seen as an attractive partner due to its role as a major donor to international health and development programs (the second largest after the United States). The choice of Germany as a location also reflected Berlin’s emergence as a regional global health and development hub.^2^ As former Gates Foundation CEO Sue Desmond-Helland explained at the Berlin office’s launch, “By establishing an office here, we hope to grow the Gates Foundation’s network across Germany and continental Europe” [53]. The Foundation also expected the new office to “allow us to tap into Germany’s thriving life sciences sector” [53].

Besides these two offices, the Gates Foundation maintains a network of representatives across the continent, who are either fully employed by the Foundation or working on a consultancy basis. This includes France, which the Foundation views France as strategically important because “it wields considerable influence over the global health and development priorities of the Group of 7 (G7) and Group of 20 (G20) nations and the European Union” [50], all actors the Foundation seeks to influence. Having a representative in Brussels is also important for engaging with the European Union, while a representative in Stockholm covers relations with the Nordic countries, which have been co-founders and among the largest donors of the Gates Foundation’s flagship initiatives, notably Gavi.

Organizationally, the “Europe team” and offices are part of the Foundation’s Global Policy and Advocacy division and are led by senior Foundation staff with high-level leadership experience from government and strategy consulting and training in political science or economics, according to their profiles on the website and on LinkedIn. London office manager Joe Cerrell has been with the Foundation since 2001, holding senior roles at the headquarters in Seattle, including as Director for Donor Government Relations and Director of Global Health Policy and Advocacy. He also set up Goalkeepers in 2017 and currently serves on the board of directors for the ONE Campaign and Global Citizen in Europe, which are both heavily supported by the Foundation. Before joining the Foundation, he served in a variety of senior roles in government and strategy consulting practices, including positions in the Clinton White House. Anja Langenbucher, who manages the Berlin office, has worked at the Foundation since 2011 and has previous experience from Boston Consulting Group (which works with the Foundation on many of its activities in Germany (e.g. [54]), and senior roles in the European Bank for Reconstruction and Development (EBRD), the IFC/World Bank, and the European Commission. Beatrice Nere, who heads the Gates Foundation’s Southern Europe, G7 & G20 relations from Paris, has worked at the Foundation since 2008, initially within the Global Health and Advocacy team in Seattle, and has experience as a Public Relations officer working on the implementation of EU legislation at the national level [55].

These executives lead Europe-based policy, advocacy and communications staff whose role is variously described as “building relationships with program partners,” “government relations,” “working to sustain European donor support for international development,” [56] “building donor support and mobilising resources” [57], and “fostering commitment to advancing global health and development goals” [53]. Specifically, the London office is described as working with “grantees and partners” to support a “constructive and well-informed political and public debate around the importance of the UK’s role in international development,” and to “advocate for the best use of the UK’s collective funding resources and its diplomatic influence in service of global health and development” [52]. Similarly, the Berlin office builds “strategic relationships with various stakeholders across Europe including governments, civil society institutions, and media” [53].

In the next section, we move beyond these broad self-descriptions and analyse what “government relations” means in practice.

### Government relations

The Gates Foundation’s European offices engage in a wide range of efforts aimed at developing and strengthening partnerships with the governments of France, Germany and the UK (as well as other key European countries and the EU), in some cases with the direct involvement of the Seattle headquarters. This is done through a range of activities including regular meetings between the Foundation’s staff and key government officials; the signing of Memoranda of Understanding (MoUs) to formalise areas of joint work; and appearances by Foundation staff (often by Bill Gates himself) at key global health and development summits, occasions which provide opportunities both for formal meetings and more informal engagements.

Foundation representatives hold regular high-level meetings with ministers and other key officials as well as routine lower-level interactions over a wide range of health and development issues. In the UK case, over four years (2020–2023), at least 37 meetings were held at Ministerial/Permanent Secretary level with Foundation representatives across four key ministries: The Foreign, Commonwealth and Development Office (FDCO) incorporating the former Department for International Development (DFID), the Department for Health and Social Care, Her Majesty's Treasury, and the Cabinet Office^3^ (see Supplementary File 1 for full details). These included eight meetings with the incumbent Prime Minister and two with the Chancellor of the Exchequer. Most often, the Foundation met with Ministers at DFID/FCDO (20 of the 37 meetings). Yet these high-level meetings are clearly only the tip of the iceberg. In 2011, Jeff Raikes, then CEO of the Gates Foundation, told the House of Commons International Development Committee that “We have a good relationship with DFID. It is a regular relationship, with regular interaction. Many of our staff will be in contact as regularly as weekly” [58].

In France, Bill and Melinda Gates met with a succession of Presidents and Ministers of Foreign Affairs and Development, totalling at least 16 times between 2010 and 2023 (see Supplementary File 1). In addition, the French lobby register specifies that the Foundation targets the President’s advisers and government officials from three ministries: Health, Foreign Affairs and International Development, and Economy and Finance. The interactions include “informal discussion,” “regular correspondence,” “events, meetings or promotional activities”, and sharing “information and expertise with an advocacy objective” [59].

Foundation staff and German officials also regularly interact [60]. Although meeting records are not available for Germany, the Gates Foundation declared having spent €4.1 million in 2023 for the representation of its interests in Germany and accredited 26 staff from both its Berlin office and headquarters in Seattle to interact with German politicians [61]. It advised the government on its global health strategy, both by sitting on the Federal Ministry of Health's International Advisory Board on Global Health in 2018 [62] and contributing with Charité and Boston Consulting to a 2019 report on Germany's leadership in global health [54]. The Foundation also co-hosts events with the German government, such as the 2018 Grand Challenges meeting in Berlin [63] and the annual World Health Summit. In 2024, Bill Gates spoke alongside, German Chancellor Olaf Scholz, WHO’s Director General Tedros Adhanom Ghebreyesus, and others at the World Health Summit’s “signature event” which aimed to support WHO’s fundraising efforts [64].

Beyond its interactions with British, French and German officials, the Foundation conducted 98 meetings with European Commission members between 2015 and June 2024, averaging ten annually. These meetings aimed to engage the Commission on a wide range of issues including health, climate, and food security, among others [65]. The Gates Foundation has also formalised its partnerships with the European governments across a range of global health and development-related areas. In the UK, the Foundation signed a 2011 ‘Strategic Partnership’ on agricultural development [66], and a ‘Collaboration Framework’ with DFID for a ‘Strategic Research Partnership’ that set out a framework through which “the Parties intend to work together more strategically on issues of common interest, and to streamline their current working relationships” [67, p1]. These are in addition to a variety of other ongoing collaborations. The 2019–20 DFID Annual Report highlighted three specific partnerships with the Foundation during that year: on reducing the costs of next-generation mosquito nets, coordinating technical working groups for the Tokyo Nutrition for Growth Summit, and launching the Ed Tech Hub [68].

A MoU with Germany’s Federal Ministry for Economic Cooperation and Development in 2017 aimed to “strengthen…collaboration on multilateral and bilateral projects under the overarching objective of significantly reducing poverty and transforming the lives of those most in need” [53]. This was built on an earlier 2011 agreement in which the Foundation pledged to match the increase in Germany’s donation to Gavi [69]. Similarly, in 2016, the Foundation signed a MoU with AFD, the French development agency, “to work together on a range of issues and across a number of regions, including collaborating on maternal, newborn, child nutrition & health and water and sanitation in West Africa” [70]. This was followed by a 2023 agreement outlining a strategic partnership on gender equality and human development across Africa and South Asia (Ibid).

Finally, the Gates Foundation actively engages with European governments through its participation in global summits and high-level international political events, such as the World Economic Forum, the Munich Security Conference, the G7, and the G20. Notably, prior to the 2011 Cannes Summit, former French President Sarkozy asked Bill Gates to prepare a report on financing for development [71]. Despite being a private actor, the Gates Foundation frequently occupies a presence and is ‘in the room’ alongside state representatives at many key global events and has long been treated by others as akin to a government, particularly at high-profile conferences addressing international aid. As Laurie Lee, then Deputy Director of the Gates Foundation, said in giving evidence to the UK House of Commons International Development Committee in 2011:

We had staff at the meeting in Paris to discuss the Paris Declaration [on Aid Effectiveness] and at that time it was not suggested that foundations - us or others - would sign it. But we were there for the discussions. We also attended the conference in 2008 in Accra [Agenda for Action] and were part of those discussions. We will be sending staff to Busan [Partnership for effective development co-operation] this month, as well. So we are very much part of discussing this [58].

### Grant-giving patterns

The previous sections examined how the Gates Foundation has developed a bureaucratic infrastructure and institutionalised its cooperation with governments in its European focus countries over the past fifteen years. In this section, we investigate how the Gates Foundation funds other societal actors within France, Germany, and the UK in ways that contribute to shaping national and international policy environments and public discourse.

From 1997^4^ to 2024, British organisations received over $3.5 billion in funding from the Gates Foundation, positioning the UK as the second largest recipient of overseas funding from the foundation. This is surpassed only by Switzerland, where most of the funding is directed towards the numerous multilateral and global organisations headquartered there. Organizations based in France and Germany were awarded $627 million and $577 million in grants, respectively, ranking them as the 9^th^ and 10^th^ largest grant recipients outside of the US (Table 1. See also supplementary material 2).

**Table 1:** Overview of the Gates Foundation’s grant-giving patterns to the UK, France, and Germany

	**UK**	**France**	**Germany**
Total value of grants directed to countries (1997–2024)	$3 552 million	$627 million	$577 million
Number of grant recipients	310	66	79
Number of grants	1399	201	189
3 single largest grantees	1) Imperial College London $338 m) 2) London School of Hygiene and Tropical Medicine ($329 m) 3) University of Oxford ($329 m)	1) French Development Agency ($206 m) 2) CGIAR System Organization ($101 m) 3) WHO’s International Agency for Research on Cancer ($47 m)	1) German Development Cooperation Agency ($163 m) 2) Evotec ($57 m) 3) DSW ($49 m)

**Fig. 1:**
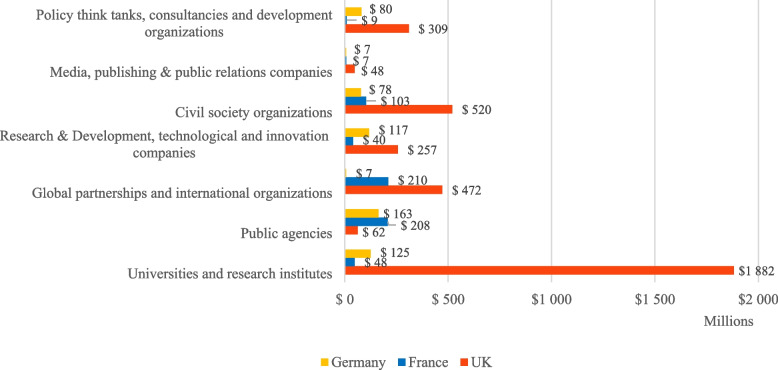
Gates Foundation’s funding to different categories of recipients in the UK, France, and Germany (USD millions)

**Fig. 2:**
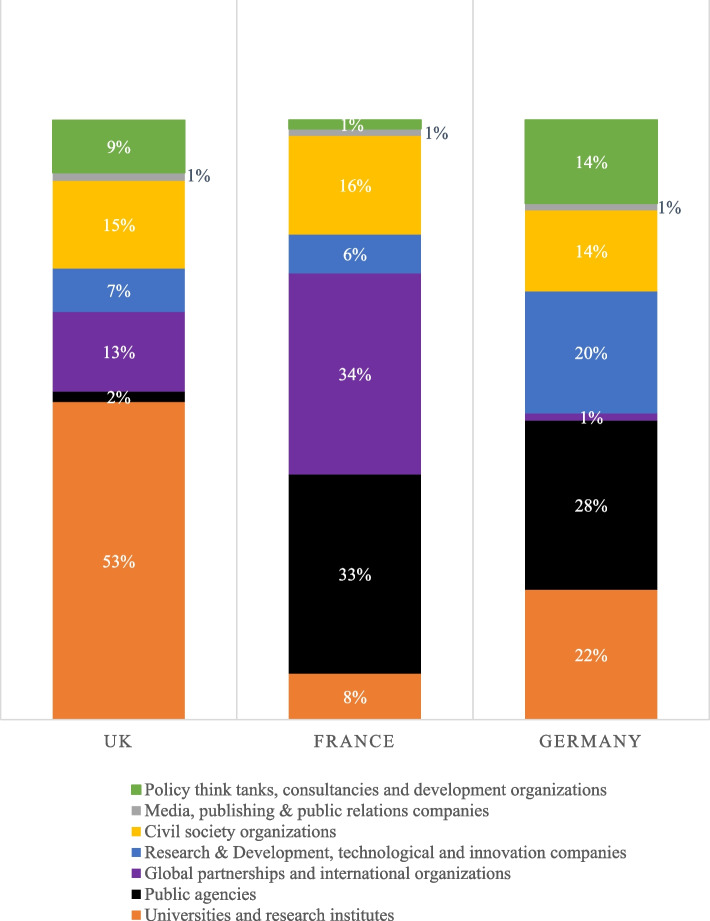
Share of the Gates Foundation’s support to different types of grantees in the UK, France and Germany

A closer examination of the list of grantees in each of the three countries highlights the wide breadth of actors supported, but also the importance of the Foundation’s spending in specific sectors (Figs. 1 and 2).

First, the Gates Foundation supports public agencies headquartered in these countries. Roughly a third of the Gates Foundation’s funding directed to France and Germany was channelled into their official Development Cooperation Agencies, making them the countries’ largest grantees. In the UK, FCDO (and formerly DFID) has not received direct funding from the Gates Foundation, although there is a significant amount of co-funding of projects between Gates and the British government. However, eleven other British public agencies, including museums, research councils, Public Health England, and the Medicines and Healthcare Products Regulatory Agency, have received over $62 million in Gates grants. Moreover, the Gates Foundation has channelled close to $700 million to multilateral organisations headquartered in France and the UK, notably to the UK-based global public–private partnerships Global Alliance for Livestock Veterinary Medicines ($189 million) and the Innovative Vector Control Consortium ($186 million) and the France-based CGIAR system organisation ($101 million), which works on agricultural innovation.

Second, the Gates Foundation has awarded large-scale grant funding to British and German innovation and research ecosystems, notably universities and research institutes. More than half of its funding to the UK ($1.9 billion) has gone to 63 British universities and research institutes. The three largest grantees, with close to $330 million each, are Imperial College London, the London School of Hygiene and Tropical Medicine, and the University of Oxford—all dominant institutions for research on global health and development. Overall, 24 British universities and research institutes have received grants totalling over $10 million. Six universities in Germany and one research institute in France have received grants of this magnitude. The Foundation has also directed some of its grants to commercial companies working on R&D and technological innovation, notably pharmaceutical and agricultural biotech companies. These grants represent a fifth of the Foundation’s funding to Germany, which is proportionally three times more than in the UK (7%) and France (6%). The largest recipients were funded to develop new diagnostics and to advance drug discovery. In addition to grants, the Foundation makes “strategic investments” in companies and other organizations “to create incentives to harness the power of private enterprise to create change for those who need it most,” which we have not analysed here [10].

Finally, the Gates Foundation has allocated over $1.16 billion to organisations based in France, Germany, and the UK working on, or promoting, development issues broadly defined. In this category, the UK stands out due to the scale of funding ($878 million) and its distribution across 159 recipients. Notably, the non-government organizations (NGOs) MSI Reproductive Choice, Save the Children, BBC Media Action and Sightsavers have received more than $50 million each, and nine other NGOs have received more than $10 million each. Some of the largest recipients are highly professionalised and international NGOs, including some who act as advocates on behalf of Gates-funded initiatives [39]. In addition, media outlets such as *The Guardian* and the *BBC*, major British policy think tanks and private research institutes working on development issues have also received substantial grants. This includes ODI ($35 million); the International Institute for Environment and Development ($33 million); the Tony Blair Institute for Global Change ($27 million); CGD Europe ($24 million); the Institute of Development Studies ($16 million); ITAD ($7 million); and Chatham House ($3 million).

A significant portion of the grants allocated to organizations focused on development issues falls under the “advocacy and policy” category as designated by the Gates Foundation. We conducted an in-depth analysis of 261 such grants allocated to its three European focus countries between 2007 and 2024 totalling over $400 million, of which the UK received the majority ($267 million), significantly surpassing Germany and France, which received $81 million and France $33 million, respectively.

The Gates Foundation began systematically investing in policy and advocacy support in the UK shortly after establishing its London office in 2010, notably by tripling its advocacy grant funding in 2012. This level of support has been maintained consistently since then. In Germany, the Gates Foundation significantly increased its advocacy grants in 2011, coinciding with the signing of its first MoU with German authorities. New commitments for advocacy support rose from $7 million in 2011 to $10 million in 2013, and to $15.5 million in 2016. A year later, a new, revised MoU was signed. French organisations did not receive substantial resources for policy and advocacy until 2018 but has since then received several grants totalling more than $24 million.

The Gates Foundation appears to support one main local NGO partner in each country through advocacy and policy grants that are both larger and longer (up to four years) than average. In the UK, this is Save the Children, which has received $59 million since 2011 to support its advocacy efforts. Save the Children employs over 140 staff in its policy and advocacy division and received $3.2 million from Gates in 2021 and 2022, corresponding to roughly one third of the division’s budget [72, p34]. In Germany, the main recipient of advocacy and policy grants is Deutsche Stiftung Weltbevoelkerung (DSW, the German Foundation for World Population), an international nonprofit foundation that supports sexual and reproductive rights and population dynamics. DSW has received a total of $46 million (between $2.4 and $3.4 million annually) for policy and advocacy since 2009. In 2021, the Gates Foundation provided more than a quarter of DSW’s total annual budget [73, p37]. Moreover, the director of the Gates Foundation’s Berlin office, Anja Langenbucher, sits on DSW’s board of trustees [73, p38]. In France, Focus 2030 is the main recipient of advocacy and policy grants. Focus 2030 was launched in 2017 as a non-profit organisation promoting the achievement of the UN’s Sustainable Development Goals by 2030, with a $833,000 Gates grant for its first four months of operation. Since 2018, Focus 2030 has received at least $1 million per year for its policy and advocacy efforts, which corresponds to more than 90% of its total annual budget [74, p30].

Although the Gates Foundation appears to have selected one main advocacy partner in each of its European focus countries, its advocacy and policy grants have been allocated to a wide range of recipients — 114 individual organisations — including NGOs, policy think tanks, media outlets, consulting groups, PR companies and universities. The largest media companies that have benefitted from a combined total of $35 million of Gates Funding for reporting on development and global health issues are *The Guardian* ($12 million between 2011–2023), followed by *Le Monde* ($6 million), *The Daily Telegraph* ($5.8 million) and *Der Spiegel* ($5.5 million), while the *BBC*, *The Bureau of Investigative Journalism*, *The Financial Times*, and *The Economist* have also received advocacy and policy grants. Numerous policy think tanks, consultancies and universities obtained policy and advocacy grants to bring together key stakeholders and organise events or develop reports and data that can be used for advocacy and set the agendas of major events.

The purpose of most of the policy and advocacy grants is formulated in broad terms, such as “to increase support for official development assistance” or “raise awareness for global health and development issues” among policymakers, media, civil society, and the general public. However, some grants are also formulated in more specific terms. ODI, for instance, received two grants worth a total of $12 million “to generate compelling and well substantiated development success stories” and “identify untold stories of sustained, macro-level development progress”.^5^ Some grants were also earmarked to support a specific policy agenda, such as “to advance the innovative development finance agenda”,^6^ “develop case studies on digital public finance”^7^ or “inform discussion around calls for a data revolution”.^8^ In some cases, the grants funded advocacy and policy linked to particular policy events, notably the World Health Summit in Berlin, the Paris Peace Forum, and the Munich Security Conference; the replenishment campaigns of the Global Fund, Gavi and the Global Financing Facility; or explicitly aimed to shape the agenda of international summits such as the G7 or G20 (see Supplementary material 2).

Although some of the activities and grants we identified can be described as political advocacy or lobbying, they comply with the Foundation’s guidelines and US law on lobbying. Private foundations registered in the US are prevented by law from lobbying the US government on specific legislation. However, there are many exceptions to this. According to the Gates Foundation own guidelines on lobbying, these exceptions include: 1) Written technical advice or assistance in response to a written invitation, 2) Nonpartisan analysis, study or research; 3) Issue advocacy addressing broad concerns; 4) Specific legislative proposals regarding matters related to jointly-funded programs (such as global partnerships); and 5) Specific legislative proposals that impact the powers, duties or tax-exempt status of the foundation (‘self-defence’ clause) [75].

## Discussion

### Summary and interpretation of findings

This study is the first of its kind to examine how the Foundation cultivates and sustains support for and continued ODA investment in its global health and development priorities among donor partners in affluent countries, notably in its European focus countries, the UK, France, and Germany. Our analysis of the Foundation's bureaucratic growth, governmental interactions, and grant allocations in these countries reveals that its role is not limited to being a “broker” between public and private stakeholders, as previously suggested by Moran [20] and further discussed by Youde and Stevenson [25]. Instead, our findings suggest that the Foundation follows a strategically crafted approach to fostering and maintaining alignment, often concealed by its frequent use of technocratic and hyperbolic discourse [76].

We find that the concept of network diplomacy offers a valuable conceptual entry-point for understanding the Gates Foundation’s activities in Europe. It helps to illuminate how seemingly disparate and disconnected practices are, in fact, integral elements of a cohesive strategy aimed at cultivating connections with governments and a diverse array of non-state actors within specific countries [11–13].

Since 2010, the Foundation has built a bureaucratic infrastructure in Europe that functions similarly to a diplomatic service [cf. 8], with country offices in London and Berlin and representatives in other strategic locations such as Paris, Brussels, and Stockholm. Our research shows that both Bill Gates and Foundation staff engage regularly with elected officials and civil servants in these countries, sometimes formalizing strategic partnerships, and exploiting key policy windows to advocate for sustained ODA contributions and support for particular objectives, such as global vaccine initiatives. These alliances are crucial for gathering both political and financial backing for joint initiatives and for leveraging European states’ diplomatic power to advance the Foundation’s policy goals within broader political fora, such as the European Union, G7, and G20. Although the Foundation’s enormous financial assets would enable it to make decisions and pursue its objectives unilaterally, it uses soft power strategies to attract or persuade governments to align with its own preferences, thus amplifying its influence and funding to its strategic initiatives.

Beyond its government relations, the Foundation’s network diplomacy includes a sophisticated public diplomacy strategy designed to maintain, consolidate, and expand its network. This strategy involves direct and indirect communication to the public and relevant stakeholders through various channels [cf. 12]. For the Gates Foundation, public diplomacy extends beyond a savvy communication and media strategy – implemented via its website and multiple social media platforms – or the celebrity influence of Bill Gates through media-focused campaigns [32, 77]. It also involves the indirect influencing of public opinion through substantial funding of civil society actors, journalists, researchers, and consultants. These organizations help communicate about global health and development projects, create new policy proposals, evaluate existing initiatives, share success stories, or advocate for ongoing ODA commitments and support for Gates-supported initiatives like Gavi, the Vaccine Alliance. Several recurrent grantees have evolved into key long-term partners for the Gates Foundation. The Foundation views these strategic investments in shaping national discourse as crucial, especially amidst rising public scepticism toward international aid [78]. Despite these efforts, ODA budgets have been slashed since 2023 in numerous major donor countries.

The Gates Foundation’s political and public diplomacy create a self-reinforcing dynamic, facilitated by its role as a central node in a complex web of formal and informal partnerships. This network is multifaceted, operating on multiple levels – local, national, regional, global – spanning various sectors [45]. From here, the Gates Foundation can exploit different channels of influence to fulfil its policy objectives, using one partnership to leverage influence over another. For example, strategic partnerships with European governments may be influenced by the Foundation’s collaborations with universities, think tanks, media, and civil society organizations, and vice versa. As Fejerskov [8] illuminated in his exploration of the Gates Foundation’s rise to power, the Foundation exercises its influence in a way that is reminiscent of a “chameleon,” sometimes acting like a state and at other times making a virtue of its non-state character to portray itself as an apolitical and independent pro-poor voice by using civil-society-inspired advocacy tactics.

The concept of network power, as outlined by Slaughter [13] and Castells [16], explains the Foundation's capacity to harness a network’s resources to rally a diverse coalition of organizations and individuals around a unified vision and collective goals. A competent bureaucracy allows the Foundation to maximize its extensive connections with both state and non-state actors, gaining expertise and culturally nuanced insights to adapt its negotiation tactics or gather support for increased influence. Prominent individuals also play an important role in the exercise of network power, as emphasized by Moon [17]. Our findings that the Gates Foundation’s executive staff hold numerous positions across multiple arenas – such as sitting on the boards of partner organisations and grantees – underscores the Foundation’s capacity to influence its networks. Finally, Grewal’s [18] account of network power suggests that as a network expands, a preferred policy option becomes dominant, appearing consensual to stakeholders and the public, thus gradually marginalizing or eliminating alternatives. Indeed, one manifestation of the Gates Foundation’s network power is that many actors coalesce around the “Gates approach” to global health, which emphasizes technology-driven, private-sector solutions focused on addressing infectious diseases in the poorest countries [42].

Nevertheless, the Foundation’s power is intertwined with the power of other network actors. Its diplomatic strategies have succeeded largely due to the presence of willing partners among European governments and the eagerness of European organisations to receive its grant funding. Alan Duncan, a former UK Secretary of State for International Development, characterised the UK government’s relationship with the Gates Foundation as a “symbiotic partnership” [58], illustrating the mutual benefits to both parties. European governments’ eagerness to collaborate with the Foundation reflects the fact that its rise coincided with the “private turn” in global governance [28]. This shift, supported by numerous European governments, involves incorporating business methodologies and new public management tactics into development aid and public policy, including through the creation of multi-stakeholder platforms and public–private partnerships. Additionally, these partnerships have aligned with government interests by amplifying the impact of their ODA funding and generating positive media coverage and public relations benefits through association with Bill Gates’ celebrity status. The concept of network power therefore reframes the debate on whether the Foundation’s power is coercive or cooperative [47], demonstrating that it can be both simultaneously.

In sum, applying the concept of network diplomacy allows us to take a holistic view of the ways in which the Foundation uses its organisational infrastructure, financial resources, and diplomatic practices in ways that together influence key European donor governments directly, and that shape the wider policy environments within which they operate.

### Limitations

This study examined a range of Gates Foundation practices by triangulating diverse sets of publicly available data. Although the Gates Foundation and the governments in its European focus countries demonstrate a certain level of transparency regarding their engagement and interactions, and despite the Foundation’s publication of a detailed grants database, the data remain incomplete and difficult to compare across countries, hindering a comprehensive portrayal of the Foundation's diplomatic practices. Crucially, available data fail to clarify the nuances of interactions between Foundation staff and national officials or reveal the contractual specifics of grants or interactions between the Foundation and its grantees. As a consequence, our understanding of the extent to which the Foundation directs, or influences decision-makers and grantees is limited. Indeed, as former Gates Foundation CEO Jeff Raikes, cited earlier, stated: “With a good intellectual dialogue, sometimes it is hard to say who influenced whom” [58].

To gain a fuller understanding of how politicians, bureaucrats, and grantees perceive their interactions with the Foundation and its representatives, in-depth qualitative research, including interviews, would be essential. However, such research faces significant challenges, primarily due to the Foundation's notorious opacity [8] and its staff’s seeming reluctance to grant interviews to researchers. This issue is further exacerbated by what Harman refers to as the “Bill Chill,” which discourages criticism of an influential actor on whom many potential informants depend for funding [32].

### Implications for future research

Our findings have important implications for future empirical research into the global expansion of the Gates Foundation’s political presence. First, more research into the Foundation’s network diplomacy in other European countries, in other world regions and in other policy domains is needed. This includes investigating the Foundation’s activities beyond liberal democracies, particularly with its “strategic partners” in China and in the Middle East, where the Foundation is actively “building partnerships with governments and private donors” [79]. Specifically, its collaboration with the Gulf Cooperation Council States, which the Foundation describes as “highly engaged in global health and development as members of key global alliances and funding mechanisms” [80], should be examined. Such partnerships may become more important in future as the US and many European donors make cuts to their overseas aid budgets.

Second, our findings highlight the necessity for in-depth research into the perspectives of the Foundation’s partners in government and civil society. Such research is needed to better understand how they seek out, respond to, accommodate and potentially resist the Foundation. Furthermore, it should examine how governments in different jurisdictions regulate private foundations, including their tax status and their transnational and national policy influence. Research is also needed into informal political norms about the role of philanthropy and other private actors in policy-making and democratic life more broadly, especially that of foreign actors. Such research can refine our findings by emphasising the agency and interests of the Foundation’s partners and grantees.

Finally, further research is warranted to explore whether other private foundations with international reach practice similar forms of network diplomacy, as well as to examine the Gates Foundation’s increasing collaboration with the philanthropic sector globally. This includes efforts aimed at encouraging other private donors to “align their giving with the priorities of the Bill & Melinda Gates Foundation” [81]. Additionally, it is important to investigate how the Foundation’s strategic investments to “stimulate private-sector driven innovation, encourage market-driven efficiencies and attract external capital to priority initiatives” [82] complement and interact with their grant-making to and strategic investments in companies through new forms of “for-profit philanthropy” [83]. This should include assessing how such investments may increase the wealth and power of private foundations and examining the potential conflicts of interest in these transactions [cf. 77].

## Conclusion

This article offers the first comprehensive study examining the practices through which the Gates Foundation operates in Europe, arguing that it practices a form of network diplomacy that enlists hundreds of actors across public, private and third sector spheres as the Foundation’s “partners” or grantees, or both. The Foundation’s network diplomacy has proven highly successful in generating significant political support for the Foundation and joint initiatives at the highest levels, including from heads of state (and royalty), and piggybacking on powerful states’ diplomatic influence on wider political alliances (EU, G20 and other donor countries etc.). Given that European countries account for approximately half of all overseas development assistance, the Foundation’s influence over how these funds are allocated has significant material consequences for the millions of people reliant on such assistance.

Furthermore, the Foundation’s grant-making, strategic investments and networking efforts have helped grow a research and innovation ecosystem across Europe that focuses on solving the Foundation’s preferred “grand challenges.” Critics argue that this approach reproduces anachronistic approaches that foreclose new policy alternatives [77]. Through its grant-making, the Foundation has successfully aligned some of Europe’s leading public health research institutions with its agenda. Finally, its strategic grant-making promotes policy analysis and public debate to generate public support for ODA, much of which is channelled through Gates-funded public–private partnerships. This includes capturing media attention on issues important to the Foundation and generating positive public relations through the grant-funded production of ‘success stories.’

Our study underscores and amplifies existing concerns regarding the influence of private philanthropic foundations over public policy and their challenge to democratic accountability and governance. These concerns include questions about the appropriateness of tax-exempt (and therefore publicly subsidised) private foundations playing an important role in public policy. Additionally, philanthropic practice has been criticised for increasing both the material wealth and political capital of donors, thus reinforcing oligopolistic corporate power and reinforcing social inequalities, while fostering dependency on charity among poor populations and countries [77, 84, 85]. The depth of engagement across society we have documented in the Foundation’s European focus countries also raises serious concerns regarding the impact of foreign private foundations on European democracies, particularly concerning the role that foreign private funding for civil society and media plays in democratic public debate. Ultimately, if everyone becomes a partner of the Foundation, who remains to hold the Foundation accountable?

## Methods

This section provides details about the methods and data we used to analyse the Gates Foundation’s network diplomacy activities. We extracted details about the Foundation's *territorial and bureaucratic presence* in Europe from the Foundation’s official website between April and September 2024 [56].

We extracted data on *government meetings* in the Gates Foundation’s European focus countries (UK, Germany and France) from a range of sources, according to country availability. For the UK, we used the published quarterly reports of official meetings held by UK Ministers and Permanent Secretaries (the most senior civil servants in each department/ministry). We examined records obtained via the relevant websites of five government departments for the years 2020 to 2023 (four years total): 1) the Department of International Development (which was closed in September 2020); 2) the Foreign, Commonwealth and Development Office (which took over responsibility for international development issues from September 2020); 3) the Department of Health and Social Care; 4) Her Majesty’s Treasury; and 5) the Cabinet Office (which includes the Office of the Prime Minister). Full listings of the meetings identified are provided in Supplementary Material 1.

Because the French and German governments do not publish comparable records, we used a variety of approaches to identify relevant official meetings. For France, we identified meetings with President Macron between 2017 and 2024 through the search function of the Elysée website [86]. We cross-checked these with Twitter/X posts from official government accounts. Although we could download the full agendas of the sitting French ministers/secretaries of states, records for their predecessors are not available. To identify such meetings, we examined the photo library of the Ministry of Foreign Affairs (which is responsible for international development issues), using the key words “Bill Gates” or “Melinda Gates” [87]. This allowed us to identify 42 pictures dating back to 2006. We used these photos and mentions to establish a list of high-level meetings between MFA officials and Bill and/or Melinda Gates. Full details of the meetings identified are provided in Supplementary Material 1.

Lobby registers were also a useful source of information on the Gates Foundation in France [88] and Germany [89], providing data about its lobbying budget, a list of Foundation staff accredited to interact with government officials and parliamentarians, details of lobbying objectives/subjects (registered each year), oversight of lobbying activities, and the category of people lobbyists seek to meet. We used additional information from the EU lobby register to triangulate findings, drawing in particular on the extensive list of meetings with Commission officials [65, 90]. Although the UK also has a lobby register [91], it does not contain useful data for our purposes as it currently only applies to consultant lobbyists, and not organisations such as the Gates Foundation.

We identified *formal partnerships/Memoranda of Understanding* between the Gates Foundation and the governments of France, Germany, and the UK by searching the official websites of relevant departments/ministries and the website of the Gates Foundation. Finally, we identified the Foundation’s *presence and participation in global health and development summits* by searching the Press releases on the websites of the Gates Foundation and of the regular global health and development-relevant gatherings including the World Economic Forum, the G7/8, the G20, the UN General Assembly, and the World Health Assembly.

These methods will not have captured all government meetings, MoUs or summit appearances. Nevertheless, for our purposes the precise numbers do not matter: our aim was to identify a regular pattern of engagement of different kinds over time, and we do not seek to make comparisons about the relative levels of engagement between our three countries of interest.

To map the Foundation’s *funding to European partners* we extracted and analysed data from the Foundation’s publicly available grant database which provides a list of over 35,000 grants committed since 1994 and includes details about the grant’s recipient, purpose, value, date committed, and duration. We downloaded the full dataset from the Gates Foundation website and extracted the entries that were awarded to recipients in France, Germany, or the UK. We coded all recipients to one of eight organisation types: universities and research institutes; technological R&D and innovation companies; civil society organisations; media, publishing and PR companies; public agencies; policy think tanks, consultancies and development organisations; global Public–Private Partnerships and International Organisations; and unclassified (for full details of the coding see Supplementary Materials 2). In a few cases, distinguishing between these categories was not straightforward, for example between think tanks and research institutes. We classified those who received around half of their Gates funding as advocacy and policy grants as think tanks.

We also undertook a more in-depth analysis of the 261 grants labelled by the Gates Foundation as “policy and advocacy” disbursed to British, French, and German grantees. We examined the identity of the grantees, the grants’ commitment dates and duration; and their stated purpose—although the vagueness of the descriptions limited the utility of this analysis. One limitation is that categories of grant (e.g. ‘policy & advocacy’) do not appear to be used consistently. Another limitation pertains to the disbursement of grants through parent organisations and intermediaries, making it impossible to fully identify all grant recipients in Europe. For instance, the Gates Foundation has awarded over $258 million to the One Campaign headquarters in Washington DC, USA, which then transfers funds to its European offices [92]. Our findings therefore likely underreport the scale of policy and advocacy grants in Europe.

## Supplementary Information

Additional file 1.

Additional file 2.

## Data Availability

No datasets were generated or analysed during the current study.

## Footnotes

1 A report commissioned by German civil society provides interesting insights into cooperation between German authorities and private philanthropic foundations—including the Gates Foundation [60]

2 Since 2009, the annual World Health Summit has made Berlin “the unique international strategic forum for global health,” providing a multi-stakeholder platform to set the agenda, with the Gates Foundation as strategic partner [64, 93]. Another core Foundation partner, the LSHTM also opened a Berlin office in 2018. It is also one of twenty-eight members of the M8 Alliance, the international network that provides the academic foundation to the World Health Summit.

3 The Cabinet Office includes the Office of the Prime Minister.

4 The Bill and Melinda Gates Foundation was officially launched in 2000, but its grant database also includes information about grants made by the William H. Gates Foundation during the period 1994 - 2000—of which three were directed to British grantees.

5 Gates Foundation [92]—Grants #OPPGD1355 and #OPP1029905.

6 Gates Foundation [92]—Grant #OPP1006743.

7 Gates Foundation [92]—Grant #INV- 064944.

8 Gates Foundation [92]—Grant #OPP1095783.
